# Crossroad between current knowledge and new perspective of diagnostic and therapy of late-onset schizophrenia and very late-onset schizophrenia-like psychosis: An update

**DOI:** 10.3389/fpsyt.2022.1025414

**Published:** 2022-10-26

**Authors:** Olga Stȩpień-Wyrobiec, Marta Nowak, Grzegorz Wyrobiec, Emilia Morawiec, Magdalena Wierzbik-Strońska, Rafał Staszkiewicz, Beniamin Oskar Grabarek

**Affiliations:** ^1^Department of Geriatrics, Faculty of Medicine in Zabrze, Academy of Silesia in Katowice, Zabrze, Poland; ^2^EMC Hospitals, John Paul II Geriatric Hospital in Katowice, Katowice, Poland; ^3^Department of Histology and Cell Pathology, Faculty of Medicine in Zabrze, Medical University of Silesia in Katowice, Zabrze, Poland; ^4^Department of Histology, Cytophysiology and Embryology, Faculty of Medicine in Zabrze, Academy of Silesia in Katowice, University of Technology, Zabrze, Poland; ^5^Department of Microbiology, Faculty of Medicine in Zabrze, Academy of Silesia in Katowice, Zabrze, Poland; ^6^Gyncentrum, Laboratory of Molecular Biology and Virology, Katowice, Poland; ^7^Faculty of Medicine in Zabrze, Academy of Silesia in Katowice, University of Technology, Zabrze, Poland; ^8^5th Military Clinical Hospital with Polyclinic - Independent Public Health Care Facility in Krakow, Kraków, Poland; ^9^Department of Gynecology and Obstetrics, Faculty of Medicine in Zabrze, Academy of Silesia in Katowice, Zabrze, Poland

**Keywords:** geriatric population, schizophrenia, cognitive impairment, antipsychotic drugs, aging

## Abstract

Schizophrenia is a chronic, highly individualized disease with many symptoms that can occur with varying severity in different patients. Schizophrenia affects 1% of the population, but occurs in almost 20% of patients after 40 years of age. It should be noted that the next peak in the incidence of schizophrenia occurs at the age of 60 years, affects mostly females, and is closely associated with a high risk of developing memory disorders. Therefore, postadolescent schizophrenia includes two distinct groups of patients: those whose symptoms onset at the age of 45 or 60. The purposes of this literature review were as follows: (1) synthetically characterize the clinical manifestations of schizophrenia; (2) discuss difficulties in the diagnosis of schizophrenia, especially in patients over 40 years of age; (3) discuss the clinical utility of different classes of marker in diagnostic and differentiating schizophrenia from neurodegenerative diseases in elderly people; (4) discuss therapeutic options for schizophrenia, pharmacotherapy, and psychotherapy, emphasizing the role of caregivers of people with psychosis in therapy, in preadolescence and postadolescence schizophrenia. We have tried to primarily discuss the findings of original articles from the last 10 years with an indication of their clinical implications with the issues discussed in the various subsections. Moreover, despite many years of research, no specific, precise algorithm has been developed that can be used in clinical practice during the diagnosis of schizophrenia. For this reason, the diagnosis of schizophrenia is primarily based on an interview with the patient and his family, as well as on the experience of a psychiatrist. It also seems that schizophrenia treatment should be carried out holistically, including pharmacotherapy, psychotherapy, and the support of caregivers of patients who have this psychosis, which increases the achievement of therapeutic success. Finally, we must be aware of the difficulties in diagnosing schizophrenia in the elderly and the need to modify pharmacological treatment. Currently, no guidelines have been developed for the differentiation of negative symptoms in elderly patients with schizophrenia from amotivation/avolition/apathy symptoms in elderly patients with neurodegenerative disorders.

## Introduction

Officially, the term “schizophrenia” was introduced as a new name for the disease entity “dementia praecox,” previously defined. The concept of “dementia praecox” was inspired by the works of Morel, who believed that psychiatric disorders result from the malfunctioning of three mental components: emotions, thinking, and behavior. In addition, he emphasized the genetic and environmental factors underlying these disorders, such as alcohol or drug addiction of parents of the sick patients (1–3). The separation of affective disorders from schizophrenia and thus, the diagnosis, was facilitated by introducing specific antipsychotic drugs into the market. In the 1950s, the first neuroleptic chlorpromazine was created, which marked the beginning of the era of psychotropic drugs. In the last two decades of the 20th century, there has been enormous progress in the neurobiology of schizophrenia due to the development of neuropathological, neuroimaging, pharmacological, biochemical, and genetic studies (4–7).

The incidence of schizophrenia is 1% of the population, but in almost 20% of patients, it appears after the age of 40 years (8, 9). According to data on the Polish population, the mean age at diagnosis is 27 years and the mean age of patients with schizophrenia is approximately 38 years (6). It is worth paying attention that the next peak in the incidence of schizophrenia falls at the age of 60 years, which concerns mainly females and is closely related to the high risk of developing memory disorders. Therefore, postadolescent schizophrenia encompasses two different groups of patients: those who develop symptoms around the age of 45 or 60 (10). Schizophrenia is a chronic, highly individualized disease with many symptoms that may occur with varying intensity in different patients. However, the proper diagnosis is made too late, especially in the elderly, and therefore, appropriate treatment is initiated too late (11).

### Clinical aspects of schizophrenia

The etiology of this disease is not entirely clear, and its underlying cause is multifactorial. In addition to genetic determinants, the role of disturbances related to dopaminergic, serotonergic, and glutaminergic transmission has been indicated, which is particularly emphasized as the underlying cause of cognitive disorders (8, 10, 12). Abnormalities in glutamatergic transmission and dysfunction of M1 and M4 muscarinic receptors significantly reduce learning opportunities (13). In the pathogenesis of schizophrenia, several factors are distinguished, such as dysregulation of the hypothalamic-pituitary-adrenal axis, accumulation of insoluble proteins, abnormal integration of white matter detected by fractional anisotropy, and a number of environmental factors, such as drug abuse, for example, amphetamines, methamphetamine, cocaine, and some behavioral factors such as physical and sexual violence, lack of family support, and loneliness, which are associated with an increased risk of suicide (8, 9, 13). Several studies have emphasized the role of interconnection between the thalamic nuclei and cortical areas (e.g., prefrontal and subcortical structures such as the hippocampus, striatum, and amygdala) in the pathogenesis of both positive and negative symptoms and cognitive disorders in schizophrenia. Furthermore, it is emphasized that the same mechanisms are at the root of negative symptoms and cognitive dysfunction, such as dysfunction of glutaminergic transmission, neuroinflammation, and oxidative stress (14, 15). Primary negative symptoms are an integral part of the schizophrenic process and are idiopathic. They are characterized by stability and chronicity, significant resistance to available therapeutic methods, and relatively low incidence, estimated at 15–25% of patients with schizophrenia (16). In clinical practice, it is difficult to distinguish and differentiate them from secondary negative symptoms. The Scale for Assessment of Negative Symptoms (SANS), developed by Nancy Andreasen, is most commonly used for this purpose (16). The scale describes five areas: shallowing of affect, alogia, avolition, apathy, attention deficit disorder, anhedonia, and unsocialization (16). This scale does not initially differentiate between the two types of negative symptoms; therefore, in research papers, the presence of a symptom in all five domains is considered the criterion for diagnosing primary negative symptoms and scoring in the range of 2–5 is considered its presence. Meanwhile, secondary negative symptoms do not arise directly from the disease process but appear due to the co-occurrence of various additional symptoms and factors associated with schizophrenia. Currently, the most important of these are considered positive (psychotic) symptoms, depression, anxiety (psychotic and non-psychotic), side effects of pharmacotherapy, addiction, and social deprivation (16). According to many authors, negative symptoms represent “what the disease took from a person and what is missing.” Symptoms progress slowly, and over time patients distance themselves from the environment and, at the same time, are open to the world of internal experiences, delusions, and hallucinations that are experienced very intensely. There is social withdrawal (autism), limited family and professional relationships, and poor form statements and content (17, 18).

However, it should be noted that the currently used methods for treating schizophrenia have three main limitations. First, their effectiveness, defined as the patient’s ability to lead an independent life, affects only approximately 50% of people with schizophrenia (19). Second, the therapeutic effect they exert is primarily directed at alleviating positive symptoms, improving only to a mild degree negative and cognitive symptoms (20). Third, their use may be associated with severe side effects leading to sexual dysfunction or agranulocytosis (clozapine). The partial effectiveness of pharmacotherapy for schizophrenia results from the pathomechanism of the disease, which is not yet fully understood (21).

## Objective

Considering the complex etiopathogenesis of schizophrenia, including the difficulty of differential diagnosis of this psychosis with neurodegenerative diseases, especially in the elderly, as well as taking into account the dynamic development of medicine, among others, including non-imaging, the search for new biochemical and molecular markers, as well as the development of personalized, predictive, preventive, and participatory (4P) medicine, the collection and presentation of current knowledge and trends in the diagnosis and therapy of schizophrenia is cognitively interesting and essential for clinical practice.

The purposes of this literature review were as follows: (1) synthetically characterize the clinical manifestations of schizophrenia; (2) discuss difficulties in the diagnosis of schizophrenia, especially in patients over 40 years of age; (3) discuss the clinical utility of different classes of marker in diagnostic and differentiating schizophrenia from neurodegenerative diseases in elderly people; (4) discuss therapeutic options for schizophrenia, pharmacotherapy, and psychotherapy, emphasizing the role of caregivers of people with psychosis in therapy, in preadolescence and postadolescence schizophrenia.

## Methodology for the collection of the reviewed data

The work we presented was classified as a “narrative literature review.” When looking for articles included in this study and critically reviewing them, we focused on the following keywords: “schizophrenia,” “marker,” “long-acting injectable medications,” “antipsychotic drugs,” “psychotherapy,” “caregiver,” and “elderly.” Therefore, given the nature of our review, there was no explicit and rigorous methodology for selecting articles, as in the case of systematic reviews. The search strategies were based on the experience of the authors of this study. In this article, we conducted an evaluation to indicate the importance of the topic we discussed and the extent to which it has been studied. We attempted to objectively present the current knowledge of the topic under discussion and base it on previously published studies. We have tried to primarily discuss the findings of original articles from the last 10 years with an indication of their clinical implications with the issues discussed in the various subsections. We searched PubMed and Google Scholar to find appropriate references. It should be noted that even in the case of a systematic review, where the selection paradigm of reports used in the target article is specified, this is not a fully objective method. Furthermore, narrative literature reviews in the case of the issue discussed in this study are justified, as it is still unclear what other more detailed questions can be posed and which can be valuably solved through a more precise systematic review (22).

## Difficulties in the diagnosis of schizophrenia, especially in patients over 40 years of age

It has been emphasized that people with schizophrenia are also more likely to suffer from several somatic diseases: myocardial infarction, arterial hypertension, chronic obstructive pulmonary disease, chronic bronchitis, emphysema, and lipid disorders, mainly hypertriglyceridemia, diabetes and lung cancer (23). There is a 30% higher 1 year mortality rate after a heart attack in people with schizophrenia compared to those without schizophrenia, mainly due to treatment discontinuation (24, 25). It is also estimated that the risk of attempting suicide in these patients is more than 12 times higher, and the overall mortality of people with schizophrenia is higher in the general population than in people with bipolar disorder, anxiety, and depression (26, 27). It is assumed that, on average, this disease is associated with an approximately 20 years shorter survival time (24).

Undoubtedly, we live in times when societies worldwide are aging gradually. Therefore, we are dealing with disease entities that previously did not occur or appeared sporadically. Polypathology and polypharmacy, closely related to old age, are important factors that hinder diagnosis and treatment, and modify the course of many diseases. The complexity of clinical situations observed in the elderly is the cause of many diagnostic and treatment difficulties observed in the geriatric population. Every disease entity may have a different, usually more complicated, and non-specific course in old age compared to the younger population. Diseases, including neurological and psychiatric disorders, deserve special attention because this group of disturbances frequently occurs, particularly in older people. Dementia, anxiety, depressive disorders, and multi-factor sleep disorders that appear *de novo* in the discussed group of patients are most common in the geriatric population. Some disease entities, such as schizophrenia, most often appear in earlier stages of life, so in geriatric age, the time of treatment reaches several, and often several dozen years (25).

The onset of clinical symptoms of schizophrenia in elderly patients is different from that observed in younger patients. In this age group, the disease mainly affects females and has a better prognosis than the early onset form (28). The presence of genetic determinants of the disease is indicated; negative symptoms of a chronic nature and cognitive impairment dominate, while positive symptoms are less frequent and less severe (28).

Late-onset schizophrenia (LOS), affecting people over 45 years of age, has a confirmed genetic predisposition, while schizophrenia onset after the age of 60 years belongs to the group of diseases known as very late-onset schizophrenia-like psychosis (VLOSLP), which is closely related to a high risk of developing neurodegenerative diseases, mainly dementia, Alzheimer’s disease, and Lewy bodies (29). Currently, very few studies have been carried out to support the hypothesis that VLOSLP is closely related to the risk of dementia; rather, it is emphasized that this group of diseases may be a harbinger of the development of dementia (30, 31). There is a high risk of misdiagnosis of schizophrenia or dementia because, in schizophrenia, memory disturbances are often predominant in the initial stage; conversely, early symptoms of dementia syndrome may suggest the presence of developing negative symptoms (29–31). In patients with VLOSLP, deficits in reasoning, attention, processing speed and remembering new information, working memory, and language function are more common, while short-term memory and visual-spatial function disorders are less severe (32). There is an average decrease in Mini–Mental State Examination (MMSE) score of one point per year over the 6 years duration of the disease, with most studies conducted in institutionalized patients, that is, those at *a priori* higher risk of cognitive impairment. Tang et al. (33) pointed out that the degree of severity of cognitive disorders, especially time perception, is positively correlated with the patient’s age, disease duration, and systolic blood pressure values.

Currently, no guidelines have been developed for the differentiation of negative symptoms in elderly patients with schizophrenia from amotivation/avolition/apathy symptoms in elderly patients with neurodegenerative disorders.

Meanwhile, in Table 1, we tried to present the main differences between LOS and dementia. Meanwhile, in the Table 2 we present key findings from studies about difficulties on schizophrenia with onset at different ages.

**TABLE 1:** Clinical characteristics of late-onset schizophrenia (LOS) vs. dementia.

Clinical feature	Late-set schizophrenia	Dementia
Sex preferences	Mostly females.	Not significant.
Premorbid personality	Some patients show predisease schizoid or paranoid personality.	No personality changes before the onset of the disease.
Delusions	Frequent present.	Relatively rare, but if they occur, there are no tools to distinguish them from delusions in schizophrenia.
Hallucinations	First and foremost, auditory.	Relatively rarely, if they occur, it is in the form of non-auditory modalities occur with a higher frequency.
Course of psychosis	Stabilization of symptoms after treatment.	Symptoms may worsen over time.
Antipsychotic response	Good.	Unknown.
Social interactions	Social withdrawal and isolation.	Behavior inappropriate to the situation.
Cognitive function	Disappearing after reaching a plateau.	Progressive.
Insight	Poor.	Poor.

**TABLE 2:** Studies on schizophrenia and the risk of developing other diseases.

References	Type of study	Results and conclusion
Correll et al. (23)	Systematic review and meta-analysis	Patients with several mental disorders, i.e., schizophrenia, have a significantly increased risk of cardiovascular disease and cardiovascular disease-related mortality, and that elevated body mass index, antipsychotic use.
Kershenbaum et al. (26)	Original research	Patients with schizophrenia have the highest risk for death compared to other groups with mental disorders. Older adults referred to an old age psychiatry service show higher rates of dementia and death than those reported for the general population.
Almeida et al. (27)	Original research	Significantly, higher risk of developing dementia in elderly males with a psychotic disorder compared to those without psychosis.
Hjorth et al. (24)	Meta-analysis	Schizophrenia is associated with a survival time of approximately 20 years shorter survival time.
Ermakov et al. (25)	Original research	Schizophrenia in patients aged ≥60 is related to the optimization of pharmacotherapy.
Huq et al. (28)	Case study	Appropriately, selected pharmacotherapy and lifestyle modification allow patients to function “relatively normally” into old age.
Bora et al. (30)	Editorial research	The diagnosis of schizophrenia in the elderly is masked by vascular changes, cognitive deficits, and atrophy of the gray matter typical of the age.
Stafford et al. (31)	Original	Patients with very late-onset schizophrenia-like psychosis (VLOSLP) represent a high-risk group for subsequent dementia due to early prodromal changes for some individuals. Factors that contributed to VLOSLP were exposure to environmental factors, including markers of deprivation, isolation, and adversity.
Yang et al. (32)	Systematic review	There is no 100% convincing evidence of a link between VLOSLP and dementia due to the small number of published studies with a large, representative population.
Tang et al. (33)	Original research	Age, illness duration, the concentration of triglycerides, and higher systolic blood pressure might be related to the cognitive deficits, respectively, in patients with schizophrenia.

## Potential markers of schizophrenia

Considering the large variation in the intensity of symptoms in individual patients, the coexistence of schizophrenia with other diseases and difficulties in the differential diagnosis of schizophrenia from other neurodegenerative diseases, research is currently being conducted to find specific markers based on which it is possible to diagnose schizophrenia quickly and without error. This is essential because, in people aged >45 years, schizophrenia coexists with other diseases, as well as some of the symptoms, such as dementia, memory impairment, and psychosis. Therefore, for this reason, predictive markers are important (29–31). Below we present current developments in finding the “ideal marker” for schizophrenia, including LOS-like psychosis and VLOSLP.

### Neuroimaging markers

The first group of markers is related to the development of non-invasive brain imaging techniques using magnetic resonance imaging (MRI).

The Consensus Report of the APA Work Group on Neuroimaging Markers of Psychiatric Disorders from 2012 indicates that so far no biomarkers of brain imaging have been developed that would have diagnostic value in psychiatry (34). The applied neuroimaging marker systems are primarily related to the diagnosis of neurocognitive disorders such as dementia. Currently, more research is needed on potential neuroimaging biomarkers in schizophrenia due to inconsistencies, small sample sizes, and the lack of multimodal studies (35).

These observations are consistent with those of Olabi et al. (36), who stated that the structural changes in the brain in the course of schizophrenia tend to progress and include white and gray matter.

Cropley et al. (37) indicated that structural changes in the brains of patients with schizophrenia depend on their age. In young people, there is a rapid loss of gray matter. Then, in middle age, this process slows significantly, and in old age the loss of white matter dominates.

In addition, Kambeitz et al. (38) showed that the sensitivity of MRI significantly depends on age, imaging modality, disease stage, while specificity depends on positive to negative symptom ratio, and antipsychotic drugs.

The most serious of these is that in patients most commonly diagnosed with schizophrenia, gray matter loss, thinning of the cerebral cortex, changes in white matter volume, and microarchitecture are, to some extent, age-related physiological phenomena (39, 40). Chung et al. (41) showed in the group of patients aged 12–17 years that the more severe neuroanatomic changes from the patient’s record age, the greater the risk of developing schizophrenia and the worse the prognosis. In turn, de Wit (42) noted that decreases in surface area of the prefrontal, cingulate, and parahippocampal are associated with less effective treatment of schizophrenia, but only among young people.

These observations indicate that the age of the patient should be considered when interpreting the neuroimaging results. Although there are specific brain neuroanatomical patterns characteristic of schizophrenia, they have certain limitations.

Table 3 summarizes the most important findings from the studies on the potential use of neuroimaging markers in schizophrenia.

**TABLE 3:** Studies on the potential use of neuroimaging markers in schizophrenia.

References	Type of study	Conclusion
Olabi et al. (36)	Meta-analysis	Patients with schizophrenia are noted to have a reduction in the volume of the entire brain, entire cerebral gray matter, frontal gray and white matter, parietal white matter, and temporal white matter, as well as a greater increase in the volume of the lateral ventricles.
Cropley et al. (37)	Original study	Patients with schizophrenia are noted with an initial rapid rate of gray matter loss that slows in middle life, followed by the emergence of a deficit in white matter that progressively worsens with age at a constant rate.
Kambeitz et al. (38)	Meta-analysis	Multidimensional pattern recognition methods are useful in identifying reliable neuroimaging-based biomarkers of schizophrenia and have 80% sensitivity and specificity.
Chung et al. (41)	Original research	Individuals at high risk of developing schizophrenia have been reported to have lower cortical volume, especially those aged 12–17. Furthermore, in this group, if there was a relapse or worsening of clinical symptoms during the 2 years follow-up period, a smaller area was found in imaging studies in the rostral part of the anterior cingulate cortex, lateral and medial prefrontal areas, and the parahippocampal cortex compared to younger subjects who had remission or stabilization of prodromal symptoms during follow-up. Worse prodromal functioning in childhood was associated with smaller areas in the medial orbitofrontal area, lateral frontal area, rostral frontal area of the cingulum, precuneus, and temporal area.
de Wit et al. (42)	Original research	It suggests that structural MRI data is valuable for quantitatively predicting long-term functional and clinical outcomes in young people with ultra-high risk of schizophrenia individuals.

SVM, support vector machine; RFE; recursive feature elimination; MRI, magnetic resonance imaging.

### Biochemical and molecular markers

Biochemical and molecular markers are the second group of markers that could be useful in differentiating schizophrenia from other conditions. This class of markers provides a chance to make a more objective and reliable diagnosis, especially when the diagnosis is uncertain based on clinical history (43).

S100 protein, nerve growth factor, and brain-derived nerve factor (BDNF) are also potentially important biomarkers of schizophrenia. Serum BDNF levels in untreated patients with schizophrenia are decreased and associated with cognitive impairment (44, 45). However, it should be noted that BDNF is not suggested as a specific marker for schizophrenia. Research suggests its role in the pathogenesis and treatment of other neurodegenerative diseases, such as Parkinson’s disease, Alzheimer’s disease, and depressive disorders (44, 45).

A blood multiset consisting of 51 markers (VeriPsych) was developed in the study by Schwarz et al. (46); however, in this case, the specificity was unsatisfactory.

Moreover, given that schizophrenia is a disease with high inheritance potential, the detection of gene expression and polymorphisms, e.g., disrupted-in-schizophrenia 1 (DISC1), is not insignificant in the context of biomarkers. However, polymorphic variants of this gene are not specific to schizophrenia. The DISC1 polymorphism has been noted, among other things, in patients with schizoaffective disorder, bipolar disorder, major depression, autism, and Asperger’s syndrome (47, 48). It has also been suggested that in schizophrenia, there is a reduction in the concentration of essential polysaturated fatty acids (EPUFAs), vitamin E, and creatinine (49), an increased concentration of asparagine, glutamine, methionine, ornithine, and taurine, and reduced levels of aspartate, glutamate, and alpha-aminoadipic acid (alpha-AAA) (50).

To date, no specific, biochemical or molecular marker has been developed that can be used in clinical practice to diagnose schizophrenia, including elderly patients.

In Table 4, we summarize studies on the possibility of using biochemical and molecular markers in schizophrenia.

**TABLE 4:** Current knowledge about the possibility of using biochemical and molecular markers in schizophrenia.

References	Type of study	Conclusion
Castrén et al. (44)	Review	A lower concentration of brain-derived neurotrophic factor (BDNF) in the brain and serum may be associated with depressive disorders.
Nagahara and Tuszynski (45)	Review	
Schwarz et al. (46)	Original research	The specificity of the blood multiset consisting of 51 markers (VeriPsych) of schizophrenia was not at a satisfactory level.
Vázquez-Bourgon et al. (47)	Original research	DISC1 gene variation may be related to the clinical severity of psychosis at the onset of the disorder.
Hennah et al. (68)	Original research	
Davison et al. (49)	Systematic review	Reduced levels of essential polyunsaturated fatty acids (EPUFAs), vitamin E, and creatinine and elevated levels of lipid peroxidation metabolites and glutamate can be observed in patients with schizophrenia.
Parksepp et al. (50)	Original research	A higher concentration of taurine and a reduced concentration of proline and alpha-aminoadipic acid (alpha-AAA) are characteristic of patients with schizophrenia who were not treated previously with antipsychotic drugs.

PRL, prolactin; FCN3, ficolin 3; APO1,2, apolipoprotein 1,2; APOC1,2, apolipoprotein C 1,2; BDNF, brain-derived neurotrophic factor; EPUFAs, essential polyunsaturated fatty acids; alpha-AAA, proline and alpha-aminoadipic acid.

### A promising machine learning

Machine learning is a promising approach to better differentiate schizophrenia from other mental disorders or neurodegenerative diseases, which is a multidimensional statistical approach to solving this problem. The combination of neuroimaging and non-imaging data significantly contributes to the predictive potential of biomarkers. In this regard, the multi-center PSYSCAN project aims to collect neuroimaging, clinical, cognitive, and genetic data and thus identify a marker useful for predicting outcomes and developing new software for data analysis in mental disorders, with particular emphasis on schizophrenia (accessed August 10, 2022)^1^ (51). The FSA score was calculated using machine learning from images obtained during functional magnetic resonance imaging (fMRI). FSA is characterized by satisfactory accuracy (80%), sensitivity (79.3%), and specificity (81.5%) in differentiating patients with schizophrenia from the control group (52). In addition, this indicator appears to be useful for predicting the response to treatment and monitoring its effectiveness (53). Table 5 shows the usefulness of machine learning in schizophrenia.

**TABLE 5:** Mechanical learning in schizophrenia.

References	Type of study	Conclusion
PSYSCAN project (51)	Internet source - database	The combination of neuroimaging data with non-imaging data from different research centers significantly increases the chances of finding a specific diagnostic and predictive marker for schizophrenia.
Li et al. (53)	Letter	Changes in function and connectivity appear to be related to schizophrenia.

## Place of pharmacotherapy and psychotherapy in late-onset schizophrenia and very late-onset schizophrenia-like psychosis

### The clinical effects of late-age schizophrenia pharmacotherapy

In general, elderly patients require lower doses of antipsychotic drugs compared to younger age groups, the principle of “start low, go slow” applies, with the starting dose of the drug being 25–50% of the dose used in young patients (54). This pharmacokinetic hypothesis may not necessarily apply to all drugs, especially to all antipsychotics; for example, during treatment with clozapine, age does not affect serum drug concentration, while smoking and male sex positively correlate with increased excretion (54). Similar observations were made for olanzapine, risperidone, and ziprasidone, in which the patient’s age did not influence the blood levels of these drugs (55). Because the active metabolite of risperidone, paliperidone, is excreted only in the urine, dose modification is required in patients with renal failure (54). Patients who do not follow medical recommendations are more often admitted to emergency psychiatric care and experience an increased number and duration of hospitalizations, which entails an increase in the costs of therapy and care for this group of patients (17). According to research, a break in the therapy of 1–10 days causes an almost 2-fold increase in the risk of disease relapse and rehospitalization (56). The risk of non-compliance in elderly patients with schizophrenia is mainly due to cognitive impairment in the first 7–10 days after hospitalization, 15–25% of patients discontinued treatment, and another crisis was observed 6 months after the disease had stabilized (56).

Hypersensitivity to antipsychotics is particularly frequent in elderly patients, which is based on an impaired blood-brain barrier and dysfunction of the P-glycoprotein, which contributes to the increased direct penetration of the drug into the central nervous system (57). Second, with age, the concentration of endogenous dopamine decreases, which leads to a decrease in the activity of the dopaminergic system as well as the synthesis of dopamine enzymes and precursors (57). Moreover, the activity of monoamine oxidase B, which is involved in the catabolism of dopamine, increases, and the concentration of the dopamine transporter responsible for its reuptake decreases (57). In the elderly population, where negative symptoms dominate, clozapine and cariprazine, a partial agonist of D3 and D2 receptors with a special affinity for D3 receptors and a partial agonist of 5-HT1A serotonin receptors, play a special role. Cariprazine is recommended as first-line monotherapy in situations where secondary negative symptoms predominate (57). In the case of intolerance, low doses of amisulpride (50–300 mg/day) are recommended, especially when negative symptoms are accompanied by depressive syndrome (57). Amisulpride has an affinity for D2 and D3 receptors and has a positive effect on symptoms such as avolition or attention deficits (57). Asenapine binds to receptors for dopamine, serotonin, histamine, and noradrenaline, showing greater efficiency in reducing negative symptoms than risperidone or haloperidol (52). A number of drugs, such as selegiline, duloxetine, citalopram, fluvoxamine, and mirtazapine, also have a beneficial effect in reducing negative symptoms. Selective serotonin reuptake inhibitors (SSRIs) reduce IL-6 and CRP levels (58). Minocycline is a second-generation tetracycline that works by reducing proinflammatory cytokines, and its effectiveness is emphasized, especially in the early and stable stages of the disease (58).

Table 6 summarizes the most important findings related to pharmacotherapy for schizophrenia, including postadolescent schizophrenia.

**TABLE 6:** Summarizing articles about schizophrenia with onset at different ages.

References	Type of study	Conclusion
Krause et al. (54)	Original research	Few clinical trials have been conducted to evaluate the efficacy and safety of antipsychotic drugs among older people with schizophrenia (excluding this study group), using a heterogeneous criterion for the definition of “older person.” Olanzapine was superior to haloperidol in overall symptoms, negative symptoms and response, and was associated with fewer dropouts than risperidone. Risperidone and haloperidol produced a more increase in prolactin than olanzapine, and olanzapine was associated with lower use of antiparkinson medication than haloperidol.
Weiden et al. (56)	Original research	According to the research, a break in the therapy of 1–10 days causes an almost 2-fold increase in the risk of disease relapse and rehospitalization. The risk of non-compliance in elderly schizophrenia patients is mainly due to cognitive impairment. In the first 7–10 days after hospitalization, approximately 15–25% of patients discontinue treatment; another crisis is observed 6 months after the disease has stabilized.
Wu et al. (57)	Review	The development of molecular biology is important to better understand the etiopathogenesis of schizophrenia and the associated negative symptoms and cognitive dysfunction. Cariprazine is a promising drug in patients with dominant negative symptoms.
Correll and Kane (52)	Viewpoint	Asenapine shows greater efficiency in reducing negative symptoms compared to risperidone or haloperidol.
Tavakoli et al. (58)	Randomized controlled trial	Selegiline, duloxetine, citalopram, fluvoxamine, or mirtazapine have a beneficial effect on reducing negative symptoms.

VLOSLP, very late-onset schizophrenia-like psychosis.

### Psychotherapy treatment in schizophrenia independent on the age

People diagnosed with schizophrenia are perceived as demanding or even difficult patients because psychotherapists experience negative emotions and sometimes do not know how to deal with such a patient. When working with people with psychosis, it is necessary to have not only a specific approach to the patient but also the therapist should have the appropriate characteristics (55, 59). Support for such individuals can be provided through supportive counseling, family interventions, cognitive-behavioral approaches, and psychodynamic psychotherapy. Psychoanalysis is a method that aims to reveal past emotional experiences of the patients and its role in influencing their current mental life. This is intended to discover the conflicts and mechanisms that precede their pathological mental state and provide guidance for psychotherapeutic management. The method of psychoanalysis is based on the use of free association, dream recall and interpretation, and interpretation of the phenomena of transference and resistance. Psychoanalysis should be conducted regularly, 3–5 times a week, for no less than 30 min, with a trained psychoanalyst (60). Furthermore, the psychoanalysis should be scheduled for at least 1 year (60). Psychoanalytic psychotherapists place a relatively high value on conceptualizing the properties of mental life (60).

Meanwhile, in psychodynamic psychotherapy, patient’s individual sessions with a psychotherapist are based on various insight-oriented, supportive or directive strategies applied flexibly (60). The main goal of psychotherapy in the case of psychosis is to support the patient with less focus on exploratory work. It focuses on defining the emotions felt by the patient, most often using techniques of clarifying, normalizing, and containing patient experiences. Psychotherapy in patients with schizophrenia occurs mainly in mainstream psychodynamic or psychoanalytical therapy (61, 62). In the psychodynamic approach, the goals are to support and provide a corrective experience, resolve emotional conflicts of the patients, and avoid situations that could cause regression (63).

Cognitive-behavioral therapy is considered the most important skill to improve the standard of social life, the acquisition of cognitive ways to cope with difficult situations, solving current problems, and developing the ability to compensate for deficits resulting from neurocognitive failure (64).

Therapists agree that working with people with schizophrenia is different from working with patients diagnosed with depression, anxiety disorders, and personality disorders. This is related to the fact that the patient-psychotherapist relationship is more intense, as well as the psychological boundaries between them. Patients with schizophrenia often experience acceptance, being heard, understood, and “accepted as they are” only in contact with the therapist (65–67). The patients themselves perceive therapy as a place where they can come and talk about difficult events, and the therapist will not be scared of it but will allow the patient to reflect on the experienced emotions. Most psychoanalysts believe that emotional problems are of primary importance for patients with schizophrenia, although they are more difficult to study systematically and empirically (68).

The main limitations of research on psychotherapy for schizophrenia are as follows: clinical heterogeneity of schizophrenia, methodological rigidity (no possibility of changing clinical management), differences in the motivation and needs of patients, differences in the possibility of using individual psychotherapy (some patients have good results, others worsen, because psychotherapy is inadequate for them; in the statistical analysis, the results cancel each other out); and finally, most patients require an integrated approach, and the study of a single treatment method does not give any insight into the possibilities of comprehensive treatment. Perhaps, researchers’ attention should be shifted from the methods to the needs of patients, and to determine what happens in effective and ineffective psychotherapy (69, 70). Therefore, controlled research should be postponed until more is known about the factors that contribute to the formation and maintenance of this therapeutic relationship (69, 70). Table 7 summarizes the most important findings related to psychotherapy for schizophrenia. Based on the available literature, there is no differentiation of psychotherapy methods in patients with schizophrenia according to age.

**TABLE 7:** Studies about psychotherapy in schizophrenia.

References	Type of study	Conclusion
Pec et al. (59)	Original research	Metacognitive reflection and insight therapy (MERIT) seems to be a promising strategy of psychotherapy in patients with schizophrenia.
Lotterman (55)	Original research	The first stage of psychotherapy in patients with psychosis should focus on recovering their ability to use language to describe their inner life.
Malmberg et al. (60)	Review	There is no reason to use psychodynamic psychotherapy techniques for hospitalized patients with schizophrenia.
Lincoln and Pedersen (61)	Meta-analysis	The form of psychotherapy for patients with schizophrenia should be individualized and take into account the psychologist and the patient.
Bjornestad et al. (62)	Original research	
Ridenour et al. (63)	Original research	Psychodynamic psychotherapy and metacognition should be considered in the treatment of patients with schizophrenia.
Sawicka and Żochowska (64)	Review	Therapy in patients with schizophrenia should focus both on overcoming the negative attitude toward one’s own disease and life and on eliminating or reducing negative symptoms.
Babl et al. (65)	Original paper	Changes in reflective functioning may serve as a common factor in psychotherapy in contrast to being a specific factor in psychodynamic therapies.
Saleh (66)	Original article	Psychotherapists should pay more attention to temperamental and character traits, levels of alexithymia, and each patient’s own defensive style.
Cheli et al. (67)	Case reports	Metacognitively oriented interventions can be recommended for schizotypal personality disorder.
Krupa and Styła (68)	Original research	Psychotherapists working with schizophrenic patients reduce the use of expressive techniques in favor of maintenance techniques.
Fonagy (69)	Meta-analysis	Patients with schizophrenia can benefit from psychodynamic psychotherapy.
Lysaker et al. (70)	Case reports	Narrative transformation should be a part of psychotherapy.

## Limitations

This study had several limitations. First, this study was not a systematic review; thus, it may have missed some relevant studies that would have been identified by systematic search. Second, in this study, although we attempted to discuss the findings presented in the original articles, we also included several review publications when, to the best of our knowledge, they contributed to the discussion of the issues raised. Third, the non-rigorous approach to the period in which the articles included in our literature review were published. Fourth, even with original works, the minimum group size necessary to include an article in our study was not considered. In addition, due to the type of the study (review article), the qualifications of the articles included in our study are probably characterized by a certain bias, as they were based on the experience of the authors of the articles.

However, it should be kept in mind, as Shrier et al. (71) noted, that even if a systematic review (with) meta-analysis is characterized by greater objectivity than other review papers, bias during interpretation does arise. Evidence-based practitioners should be aware that conclusions and recommendations based on a systematic review with a meta-analysis should be read cautiously, even if the methodology is rigorous (71).

## Conclusion

Although the term schizophrenia was defined in 1893, research is still being carried out on the optimization of the pharmacotherapy used in the treatment of this psychosis based on individual and population variability following the principle of “personalization of medicine.” In addition, the search for “ideal” biomarkers of schizophrenia, based on specific structural and dynamic changes in the brains of people with schizophrenia biochemical and molecular markers, is constantly being sought. However, to date, nothing can replace the experience of a psychiatrist and a thorough interview with the patients and their families. This review also draws attention to the fact that therapy should be holistic, not only including pharmacological treatment but also psychotherapy, and considers the key role of caregivers for people with schizophrenia. It is worth mentioning that the most important aspect is to diagnose schizophrenia as early as possible and thus start appropriate treatment as soon as possible. Unfortunately, we often deal with situations where schizophrenia is diagnosed several years after the onset of symptoms when the severity of symptoms and the decline in the patient’s functioning in society are already significant (40). There are several possible reasons for this finding. First, as indicated in this study, the “ideal” marker of schizophrenia has yet to be identified. Second, physicians’ experience in recognizing symptoms and the low public awareness of symptoms are factors of great importance. Third, schizophrenia, especially in the elderly, is relatively rare and its symptoms, especially the negative ones, may be confused with amotivation/avolition/apathy symptoms accompanying neurodegenerative disorders, which are common in old age.

**FIGURE 1:**
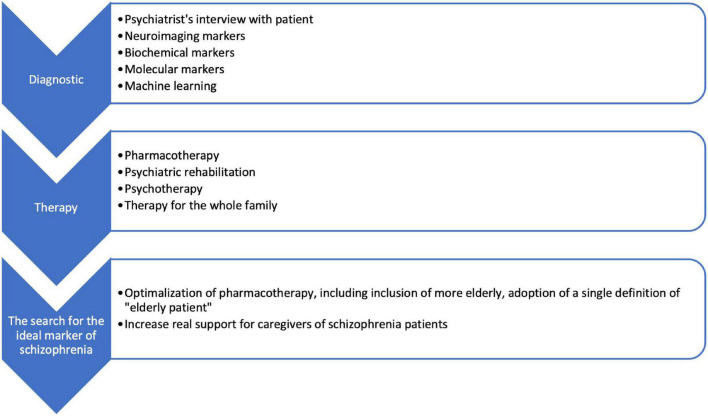
Challenges in diagnosis, treatment of schizophrenia, and indicated next steps.

However, studies on the search for “ideal biomarkers” for prognostic, diagnostic, predictive, and optimizing treatment of schizophrenia should not be stopped because this is supported by progress in medicine and related sciences. In Figure 1, we have included the most important challenges identified in this study regarding the diagnosis and treatment of schizophrenia and the following steps.

## Author contributions

OS-W and MN: conceptualization. MW-S: formal analysis. OS-W, MN, and GW: writing – original draft preparation. EM and RS: writing – review and editing. OS-W and BG: supervision. All authors have read and agreed to the published version of the manuscript.

## Footnotes

1
www.psyscan.eu
